# Redox Systemic Signaling and Induced Tolerance Responses During Soybean–*Bradyrhizobium japonicum* Interaction: Involvement of Nod Factor Receptor and Autoregulation of Nodulation

**DOI:** 10.3389/fpls.2019.00141

**Published:** 2019-02-15

**Authors:** Tadeo F. Fernandez-Göbel, Rocío Deanna, Nacira B. Muñoz, Germán Robert, Sebastian Asurmendi, Ramiro Lascano

**Affiliations:** ^1^Instituto de Fisiología y Recursos Genéticos Vegetales, Centro de Investigaciones Agropecuarias, Instituto Nacional de Tecnología Agropecuaria, Córdoba, Argentina; ^2^Departamento de Ciencias Farmacéuticas, Facultad de Ciencias Químicas, Instituto Multidisciplinario de Biología Vegetal, Universidad Nacional de Córdoba, Consejo Nacional de Investigaciones Científicas y Técnicas, Córdoba, Argentina; ^3^Cátedra de Fisiología Vegetal, Facultad de Ciencias Exactas, Físicas y Naturales, Universidad Nacional de Córdoba, Córdoba, Argentina; ^4^Instituto de Biotecnología, Centro de Investigaciones en Ciencias Veterinarias y Agronómicas, Instituto Nacional de Tecnología Agropecuaria, Buenos Aires, Argentina

**Keywords:** systemic changes, redox signaling, rhizobia, soybean symbiosis, ISR/PGPR-like response, autoregulation of nodulation

## Abstract

The symbiotic relationship between legumes and nitrogen-fixing rhizobia induces local and systemic responses, which ultimately lead to nodule formation. The autoregulation of nodulation (AON) is a systemic mechanism related to innate immunity that controls nodule development and involves different components ranging from hormones, peptides, receptors to small RNAs. Here, we characterized a rapid systemic redox changes induced during soybean–*Bradyrhizobium japonicum* symbiotic interaction. A transient peak of reactive oxygen species (ROS) generation was found in soybean leaves after 30 min of root inoculation with *B. japonicum*. The ROS response was accompanied by changes in the redox state of glutathione and by activation of antioxidant enzymes. Moreover, the ROS peak and antioxidant enzyme activation were abolished in leaves by the addition, in either root or leaf, of DPI, an NADPH oxidase inhibitor. Likewise, these systemic redox changes primed the plant increasing its tolerance to photooxidative stress. With the use of non-nodulating *nfr5*-mutant and hyper-nodulating *nark*-mutant soybean plants, we subsequently studied the systemic redox changes. The *nfr5-*mutant lacked the systemic redox changes after inoculation, whereas the *nark-*mutant showed a similar redox systemic signaling than the *wild type* plants. However, neither *nfr5-* nor *nark-*mutant exhibited tolerance to photooxidative stress condition. Altogether, these results demonstrated that (i) the early redox systemic signaling during symbiotic interaction depends on a Nod factor receptor, and that (ii) the induced tolerance response depends on the AON mechanisms.

## Introduction

The symbiotic interaction between legume plants and nitrogen-fixing soil bacteria has great importance at a basic, ecological and economic level and, the legume–rhizobium symbiosis interaction is the most important in terms of the biological nitrogen fixation (Graham and Vance, 2003). Moreover, the legume–rhizobium interaction could also induce PGPR like-responses, improving host plant growth and tolerance/resistance to abiotic/biotic stress conditions. In this sense, it has been postulated that the enhanced tolerance/resistance in inoculated plants is achieved by an ISR modulated by JA and ET (Pieterse et al., 2014).

Rhizobia have acquired the ability to evade the initial microbe-associated molecular pattern (MAMP) triggered immunity (Boller and Felix, 2009) by modulating the host immune response to avoid being recognized as a pathogen (Zamioudis and Pieterse, 2012). In this regard, many biochemical, molecular and hormonal changes occur at local and systemic levels during the legume–rhizobium interaction so that rhizobia coordinate the organogenesis of the nodule with infection (Nadzieja et al., 2018).

The species-specific interaction between rhizobia and their host plant is determined by nodulation (Nod) factors from rhizobia and the structure of the Nod-factor receptor (NFR) in the host. Nod factors are formed by a chitin backbone with an N-linked fatty acid moiety attached to the non-reducing terminal sugar and other modifications (Oldroyd and Downie, 2008; Oldroyd et al., 2011).

Nitrogen availability and Nod factors are major local regulators. On the other hand, an innate immunity-related mechanism, called AON, is the systemic control of the nodulation process and involves long-distance root–shoot–root signaling (Caetano-Anolles and Gresshoff, 1991; Ferguson et al., 2010, 2018). During AON, rhizobia trigger the synthesis of CLE peptides in the root (GmRIC1 and GmRIC2 in soybean) that move via xylem to the shoot, where they bind to a LRR receptor kinase, the nodulation autoregulation receptor kinase (NARK). This binding triggers the production of a SDI, which moves back to the roots and inhibits nodule formation (Searle et al., 2003; Okamoto et al., 2009, 2013; Ferguson et al., 2010, 2018; Indrasumunar et al., 2011; Reid et al., 2013). Although the chemical nature of SDI is not completely known, some evidence postulates CKs as putative SDI (Chen et al., 2014; Sasaki et al., 2014; Mens et al., 2018).

Reactive oxygen species were initially characterized as toxic by-products of aerobic metabolism. Nowadays, however, ROS are also considered signaling molecules involved in several signaling pathways in organisms from bacteria to mammal. Thus, the concept of oxidative stress moves toward oxidative or redox signaling. This dual role of ROS depends on its tight generation/scavenging ratio in different subcellular location. The ROS scavenging capacity of plants is supported by the well-known antioxidant system, which is composed by soluble antioxidants like ascorbate and glutathione as well as by enzymes like SODs, CATs, APXs, and GRs. This system forms a hub that acts as a cellular redox state buffering mechanism that regulates the cellular redox homeostasis. Changes of the cellular redox state provide important information and act on the sensing, signaling and response to internal or external stimuli (Asada, 1999; Foyer and Noctor, 2009).

The RBOH proteins or NADPH oxidase complex in plants are major sources of apoplastic ROS and key players in the oxidative or redox signaling. RBOH mediates cell-to-cell communication and long-distance signaling in response to different stress conditions. The long-distance or systemic signaling mediated by ROS travels at a rate similar to an electric signal, and is independent of ET, JA, or SA signaling. The systemic redox signaling participates in the regulation of systemic acclimatory mechanism to stress conditions (Miller et al., 2009). ROS and nitric oxide are involved in the systemic defense signals, either against pathogens or abiotic stress, as evidenced by their synthesis upon these stresses, and can be propagated over long distances through the phloem (Gaupels et al., 2017).

Local redox changes in root hairs and roots induced during different stages of the symbiotic interaction have also a key role in nodulation regulation (Peleg-Grossman et al., 2007; Cárdenas et al., 2008; Muñoz et al., 2012, 2014b; Robert et al., 2014, 2018; Arthikala et al., 2017).

In this study, we analyzed the systemic redox changes during the *Glycine max–Bradyrhizobium japonicum* interaction and their implications on ISR/PGPR-like response. In addition, we investigated the involvement of Nod factors perception and AON mechanisms in the ISR/PGPR-like response by performing experiments with non-nodulating *nfr5-* and hyper-nodulating *nark*-soybean mutants. Thus, the feature of AON as an autoimmunity mechanism as well as the involvement of the anti-senescence hormone CKs in the systemic control of nodule development led us to hypothesize that AON mechanisms are underlying the ISR/PGPR-like response induced during legume–rhizobia symbiotic interaction.

## Materials and Methods

### Plant and Bacterial Growth Conditions

*Glycine max* (L.) Merr. *cv.* Bragg *wild type (wt), nts1007* (hyper nodulating *nark-*mutant) and *nod139* (non-nodulating *nfr5-*mutant) (Carroll et al., 1985, 1986) were used in this work. Seeds were surface-disinfected for 10 min in sodium hypochlorite 5% (v/v) and washed successively with distilled water. The seeds were germinated on filter paper moistened with distilled water in a chamber at 28°C in the dark for 3 days. Then, seedlings were placed in aerated plastic trays with B&D solution (Broughton and Dilworth, 1971) supplemented with 0.625 mM KNO_3_ and 0.313 mM Ca(NO_3_)_2_ (1,25 mM of total nitrogen concentration). The B&D solution was replaced every 7 days. Soybean plants were grown in a growth chamber under 16 h photoperiod (250 μmol m^-2^ s^-1^) at 26 ± 2°C.

*Bradyrhizobium japonicum* USDA 138 strain was cultured in yeast extract mannitol (YEM) medium (Vincent, 1971) at 28°C with constant agitation (180 rpm) for 5 days. For the treatments, the bacteria were washed and resuspended in sterile water (OD_600_ = 0.8).

### Treatments Conditions

The root of 12-days-old soybean plants were inoculated with 2.5 mL of washed *B. japonicum* USDA 138 strain (OD_600_ = 0.8) per liter of hydroponic medium. The first trifoliate leaf was collected at different times post-inoculation and immediately frozen or used for measurement.

For the DPI treatments, an NADPH-oxidase inhibitor (50 μM), was added 30 min before inoculating *B. japonicum* to either the root or leaf according to the experiment. The inhibitor was added to the hydroponic medium for the root application or sprinkled locally in the first trifoliate leaf. DPI application does not affect the normal growth of *B. japonicum* (Muñoz et al., 2012).

For the photooxidative stress treatment, the first trifoliate leaf of 12-days-old inoculated and non-inoculated (control) plants were treated with 1,1′-dimethyl-4, 4′-bipyridinium dichloride (PQ). PQ is a viologen that acts at the photosystem I level by intercepting the electrons that are going from ferredoxin to NADP+, thus enhancing chloroplastic ROS generation and inhibiting ascorbate regeneration (Asada, 1999). The leaves were splashed uniformly with 20 μM of PQ and 0.01% Tween 20 at 24 and 48 hpi and physiological parameters were measured after 24 h of PQ treatments. PQ was replaced by water in control treatments.

Purified Nod factors and chitosan treatments were performed in the same way than *B. japonicum* inoculation, in the hydroponic medium. Chitosan is a fungal elicitor.

### Apoplastic Superoxide Radical (O_2_^-^) and Hydrogen Peroxide (H_2_O_2_) Production in Leaves

Superoxide levels were determined histochemically with nitroblue tetrazolium (NBT) staining, which reacts with superoxide radicals to produce a blue formazan precipitate. Leaves were incubated in 0.01% (w/v) NBT solution in 25 mM K-Hepes buffer (pH 7.6) in the darkness at 28°C for 2 h. Color images were transformed in invert 8-bit images and gray value intensity of the blue stain was quantified using the ImageJ software (Schneider et al., 2012).

H_2_O_2_ generation was histochemically determined with 3,3′-diaminobenzidine (DAB) staining. The leaves were incubated in 0.02% (w/v) DAB solution in 50 mM Tris acetate buffer (pH 5) and incubated in the darkness at 28°C for 2 h. Color images were transformed into invert 8-bit images and gray value intensity of the DAB stain was quantified using Optimas 6 (Optimas Corporation, Bothell, WA, United States). Total optical density (OD) was calculated as log inverse gray value of pixels within an area boundary relative to the analyzed area.

Hydrogen peroxide was also estimated in leaf extracts as previously described by Guilbault et al. (1968). A blank with CAT was included for each sample. Frozen leaf samples were ground to a fine powder with liquid nitrogen and homogenized 1/10 (w/v) in 50 mM potassium phosphate buffer (pH 7.5), containing 1 mM EDTA and 1% polyvinylpolypyrrolidone (PVPP). Homogenates were centrifuged at 16,000 × *g* at 4°C for 25 min and the supernatant was used to determine protein and hydrogen peroxide concentrations.

### Glutathione and Ascorbate Content

The glutathione and ascorbate content in soybean leaves were determined as previously described by our group (Rodríguez et al., 2010). Leaf samples were homogenized (100 mg of fresh weight material) in 1 mL of cold 3% trichloroacetic acid (TCA) and 100 mg PVPP. The homogenate was centrifuged at 10,000 × *g* at 4°C for 15 min and the supernatant was collected to for analyze of glutathione and ascorbate content.

### Antioxidant Enzymatic Activities

Superoxide dismutase activity was determined spectrophotometrically at 560 nm, as previously described by Casano et al. (1997). GR activity was assayed following the decrease at A340 nm because of NADPH oxidation, according to Schaedle and Bassham (1977). CAT activity was determined by measuring the decrease at 240 nm because of H_2_O_2_ degradation (Gallego et al., 1996). APX activity was measured according to Nakano and Asada (1981) by measuring the H_2_O_2_-dependent oxidation of ascorbate at 290 nm (the extract medium for this enzyme contained 5 mM ascorbate).

### Total Protein Content

Soluble proteins were estimated according to Bradford (1976). Bovine serum albumin was used as a standard for calibration.

### Chlorophyll Content

The Chlorophyll content of the first trifoliate leaf was estimated using a handheld SPAD CL01 meter (Hansatech Instruments, Pentney King’s Lynn, United Kingdom). The results are expressed as unitless parameter (SPAD units, 0 to 100), which is proportional to leaf chlorophyll.

### Chlorophyll Fluorescence and Photosynthesis

Quantum efficiency of PSII photochemistry under ambient light conditions (250 μmol photon m^-2^ s^-1^, 25 ± 2°C) (ΦPSII) was measured using a pulse amplitude modulated fluorometer (FMS2, Hansatech Instruments, Pentney King’s Lynn, United Kingdom). *F*_v_/*F*_m_ ratio was calculated using (*F*_m_ -*F*_0_)/*F*_m_, where *F*_m_ is maximal fluorescence yield of the dark-adapted state and *F*_0_ is minimum fluorescence yield (Maxwell and Johnson, 2000).

Photosynthesis was determined using a portable infrared gas analyzer (Li-COR-6400, United States). Leaf chamber setting parameters were: LED light source of 800 μmol. m^-2^ s^-1^, leaf temperature 25–26°C, CO_2_ concentration of 400 μmol. mol^-1^ and 500 flow.

### Performance Index

The performance index, is an indicator of leaf vitality, the measurements were performed with the Pocket PEA chlorophyll fluorimeter (Hansatech Instruments, Pentney King’s Lynn, United Kingdom).

### Total Antioxidant Capacity (FRAP), Malondialdehyde Content (MDA), Chlorophylls, and Sugars

The samples were homogenized using a mortar and pestle under liquid nitrogen and adding 80% EtOH. Then, centrifugation was carried out at 12,000 × *g*, 4°C during 10 min. This extracts were used to measure FRAP, lipid peroxidation (MDA content), chlorophylls and sugars.

For FRAP determination, 5 μL of supernatant was diluted 20 times with 80% EtOH and mixed with 100 μL reaction buffer (5 mL of acetate buffer 0.3 M pH 3.6; 0.5 mL of TPTZ 10 mM (2,4, 6 Tris (2 pyridyl) s-triazine) diluted in 40 mM HCl and 0.5 mL of FeCl_3_ 200 mM). This mixture was left to stand for 20 min at room temperature to react and the reaction was subsequently measured at 593 nm. TROLOX was used as a standard to calculate FRAP capacity of the samples (Benzie and Strain, 1996).

Malondialdehyde levels were quantified according to Heath and Packer (1968). Briefly, 200 μL of each sample was mixed with 200 μL of 20% TCA + 0.5% TBA, incubated at 80°C for 20 min and immediately cooled in ice. The mix was centrifuged at 13,000 × *g* for 10 min and the absorbance of the supernatant was read at 532 nm and 600 nm.

Chlorophylls were calculated by spectrofluorometry at 654 nm in a reaction with 50 μL of extract and 450 μL of EtOH.

Sugars were determine in a reaction of 5 μL of each extract with 45 μL of EtOH and 150 μL of antrone that was incubated 20 min at 4°C, 30 min at 80°C and 20 min at 25°C. The reactions were subsequently measured at 620 nm. Glucose was used to calibrate a standard curve.

### Phenylalanine Ammonia-Lyase Activity (PAL)

The PAL activity was measure according to Beaudoin-Eagan and Thorpe (1985). The enzyme activity was expressed in nmol of *trans*-cinnamic acid.mg protein^-1^.min^-1^, where 1 U is defined as 1 nmol *trans*-cinnamic acid.mg protein^-1^.min^-1^. 300 mg of leaf samples were homogenized in 3 mL of Tris-HCl buffer 50 mM pH 8.5 with 2-mercaptoethanol 14.4 mM and 100 mg PVPP, and centrifuged at 6,000 × *g* for 10 min at 4°C. The supernatant were passed through a column of celite and centrifuged at 10,000 × *g* for 15 min at 4°C. Total protein concentration was determined using the Bradford (1976) assay.

### Hormone Identification and Quantification by Liquid Chromatography Electrospray Ionization Tandem Mass Spectrometry (LC-ESI/MS-MS)

Phytohormones were extracted from 200 g of dry weight leaf as previously described (Durgbanshi et al., 2005) with some modifications. Plant material was homogenized in an ultraturrax T25 basic homogenizer (IKA, Staufen; Germany) with 5 mL deionized water. D2-SA and D6-JA (Leibniz- Institute of Plant Biochemistry; Halle, Germany) were used as internal standards. A total of 50 ng of each standard was added to the samples. The samples were centrifuged at 1,540 × *g* for 15 min and the supernatant was adjusted to pH 2.8 with 15% (v/v) acetic acid and extracted twice with diethyl ether. The organic fraction was evaporated under vacuum. Dried extracts were dissolved in 1 mL methanol and filtered on a vacuum manifold at a flow rate below 1 mL.min^-1^. The eluate was evaporated at 35°C under vacuum in SpeedVac SC110 (Savant Instruments; New York, NY, United States). Four biological replicates were used for the assays.

Hormones were separated from samples using reversed-phase high-performance liquid chromatography (HPLC). An Alliance 2695 separation module (Waters; Milford, MA, United States) equipped with a Resteck Ultra C18 column (100 × 2.1 mm, 3 μm) was used to maintain performance of the analytical column. Fractions were separated using a gradient of increasing methanol concentration, constant glacial acetic acid concentration (0.2% in water), and an initial flow rate of 0.2 mL min^-1^. The gradient was increased linearly from 40% methanol/60% water-acetic acid at 25 min to 80% methanol/20% water-acetic acid. After 1 min, the initial conditions were restored, and the system was allowed to equilibrate for 7 min. Hormones were identified and quantified using a quadruple tandem mass spectrometer (Quattro Ultima, Micromass; Manchester, United Kingdom) fitted with an electrospray ion (ESI) source, in multiple reactions monitoring mode (MRM). This assay was performed using precursor ions and their transitions (m/z) to SA (m/z 137/93), D2-SA (m/z 141/97) and JA (m/z 209/59), D6-JA (m/z 215/59) with retention times of 4.35 and 14.30 min, respectively. Collision energies used were 20 eV for both and the cone voltage was 35 V. The analyses were accomplished using MassLynk version 4.1 (Micromass).

### Ethylene Content by Gas Chromatography

Prior to *B. japonicum* inoculation, 12-days-old plants were transferred to 580 cm^3^ glass jars containing 100 mL of B&D solution. Three plants per bottle were placed and inoculated with *B. japonicum.* The containers were sealed to prevent the loss of gaseous hormone. Subsequently, samples were taken through syringes of 10 mL 30 min post inoculation, and subsequently conserved in sealed 10 cm^3^ vial tubes at 4°C until quantification.

Ethylene content was determined by gas chromatography according to adaptations of the methodology described by Hardy et al. (1968). Briefly, 1 mL of air sample was injected and quantification was carried out for 2 min in a Hewlett Packard Series II 5890 gas chromatograph by determining the ET peak between the retention times of 1.4 and 1.55 min.

### Statistical Analysis

Three independent experiments with their corresponding number of replicates for each case were performed in different dates. All data are presented as the mean ± standard error (SE). Comparisons between different treatments were analyzed by ANOVA and Tukey tests using InfoStatTM Software (Di Rienzo et al., 2016). The results were considered significant when *p* < 0.05.

A multivariate statistical analysis was performed using a principal component analysis (PCA) (Johnson and Wichern, 2006) to explore associations between genotypes/treatments and the set of physiological variables.

## Results

### NADPH Oxidase-Dependent Systemic Redox Changes Are Induced in Soybean Leaves After Root Inoculation With *Bradyrhizobium japonicum*

**FIGURE 1:**
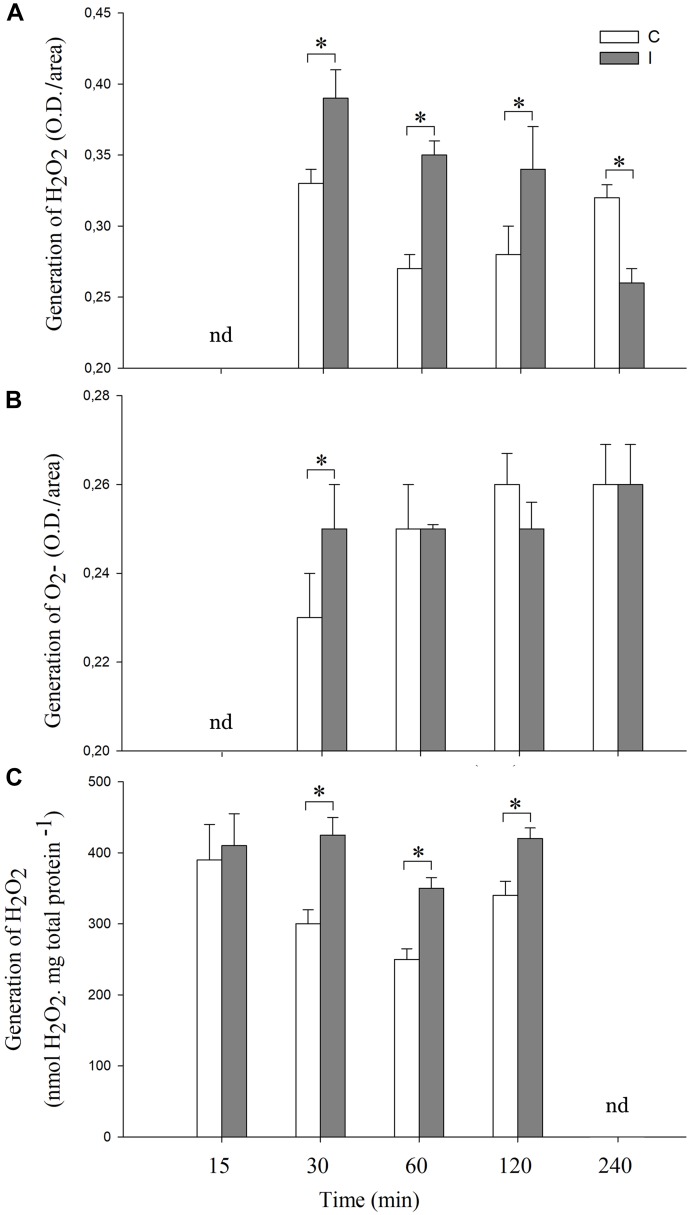
Systemic ROS generation is induced after root inoculation with *B. japonicum*. Leaves of non-inoculated (control, C) and inoculated (I) plants were sampled at different times after root inoculation (nd represent times not determined), and incubated with **(A)** 3,3′-diaminobenzidine (DAB) or **(B)** NBT for H_2_O_2_ and O_2_^-^ staining, respectively. The pictures of the stained leaves were transformed into 8-bit images and the gray value intensity (O.D.) was quantified by image processing software. Results are the means of three independent experiments (four leaves per treatment). Data are means ± SE. Asterisks indicate significant difference between control and inoculated treatments (*p* < 0.05, Tukey test). **(C)** H_2_O_2_ quantification by spectrofluorometer. The results are the means of three independent experiments (six leaves per treatment). Data are means ± SE. Asterisks indicate significant difference between control and inoculated treatments (*p* < 0.05, Tukey test).

As mentioned above, the establishment of the symbiotic relationship between legumes and rhizobia depends on both local and systemic complex signaling pathways (Ferguson et al., 2018). In this regard, although the participation of local ROS signaling in roots after symbiont perception has been widely studied (Cárdenas et al., 2008; Muñoz et al., 2012, 2014a; Damiani et al., 2016), much less is known about the ROS systemic production during the legume–rhizobium interaction. We evaluated histochemically the systemic ROS (O_2_^-^ and H_2_O_2_) generation in leaves after 30, 60, 120 and 240 min of root inoculation with *B. japonicum* (Figure 1A,B). The pictures of the stained leaves were transformed in numeric values using image processing software as described in “Materials and Methods.” Besides, the content of H_2_O_2_ in leaves was also quantified by spectrofluorometer (Figure 1C). Interestingly, the H_2_O_2_ and O_2_^-^ generation were differentially modulated in leaves after root inoculation (Figure 1). The inoculated plants displayed H_2_O_2_ generation from 30 to 120 min after root inoculation and the levels were higher than those of the control plants (Figure 1A,C). However, these levels decreased after 240 min post inoculation even below the levels of H_2_O_2_ observed in the control plants (Figure 1A).

In the case of O_2_^-^, a transient peak of O_2_^-^ generation was induced at 30 min post inoculation, whereas no differences were observed in the later times with respect to the non-inoculated control plants (Figure 1B). Moreover, this systemic and transient response was specific for *B. japonicum*. Indeed, the addition of purified Nod factors to the roots induced a transient systemic ROS induction, whereas treatments with chitosan, a fungal elicitor, induced a sustained systemic ROS induction (Supplementary Figure S1).

**FIGURE 2:**
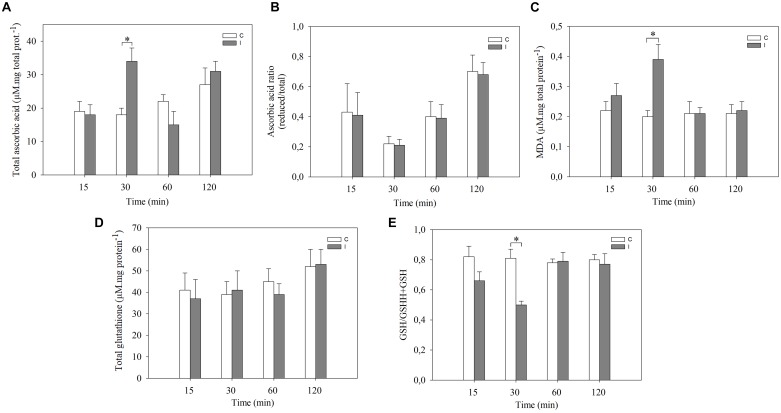
Systemic redox changes are observed after root inoculation with *B. japonicum*. Leaves of non-inoculated (control, C) and inoculated (I) plants were sampled at different times after root inoculation and **(A)** total ascorbic acid, and **(D)** glutathione levels were measured. The reduced/total ratio of **(B)** ascorbic acid, and **(E)** glutathione was calculated. MDA content **(C)** was quantified. Data are means ± SE of three independent experiments. Asterisks indicate significant difference between control and inoculated treatments (*p* < 0.05, Tukey test).

**FIGURE 3:**
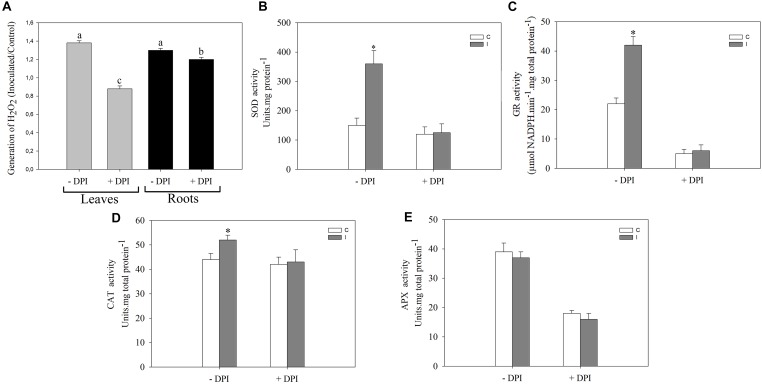
NADPH oxidase complex modulates the systemic redox changes after root inoculation with *B. japonicum*. Leaves or roots of 12-days-old soybean plants were pretreated 30 min with 50 μM DPI (+DPI) or DMSO (–DPI), and then roots were inoculated with *B. japonicum*. After 30 min of root inoculation, leaves of non-inoculated (control, C) and inoculated (I) plants were sampled and incubated with **(A)** 3,3′-diaminobenzidine (DAB) for H_2_O_2_ quantification. The results are expressed relative to non-inoculated plants (assigned a value of 1). DAB precipitated in leaves was measured and transformed into optical density (OD) by the image processing software Optimas^®^. The results are the means of three independent experiments (four leaves per treatment). Different letters indicate significant differences between the treatments (*p* < 0.05, Tukey test). SOD **(B)**, GR **(C)**, CAT **(D)** and APX **(E)** activities were measured 30 min post inoculation in leaves of plants whose roots were pretreated with DPI (+DPI) or DMSO (–DPI). Data are means ± SE of three independent experiments. Asterisks indicate significant difference between control and inoculated treatments (*p* < 0.05, Tukey test).

In order to further characterize the cellular redox state changes in leaves after root inoculation, the content of ascorbic acid, glutathione, MDA and the activities of SOD, GR, CAT, and APX were measured (Figure 2, 3).

The total ascorbic acid content increased in leaves only at 30 min post inoculation compared to leaves of non-inoculated plants (Figure 2A). No significant differences were observed in the ascorbic acid redox state (reduced/total ratio) at any of the evaluated times (Figure 2B). On the other hand, total glutathione content in leaves remained unaltered with the inoculation. The glutathione redox state (reduced/total ratio), however, decreased at 30 min in leaves of inoculated plants compared with control plants (Figure 2D,E). Likewise, the content of MDA, which is an intermediary metabolite of lipid peroxidation used as an oxidative stress marker, significantly increased in leaves of inoculated plants after 30 min of root inoculation thereby correlating with ROS induction (Figure 2C).

NADPH oxidase complex plays important roles in local signaling during symbiotic interaction (Peleg-Grossman et al., 2007; Cárdenas et al., 2008; Montiel et al., 2012; Muñoz et al., 2012; Robert et al., 2018) as well as in systemic signaling triggered by wounding, heat, cold, high-intensity light, and salinity stress (Miller et al., 2009). To investigate the participation of this complex during the systemic ROS induction in leaves after root inoculation, we pretreated the roots or leaves with 50 μM DPI, a well-known inhibitor of flavoprotein enzymes that is extensively used as an NADPH oxidase inhibitor (Figure 3). Interestingly, the induced systemic ROS generation observed in leaves of inoculated plants was completely abolished when DPI was applied in leaf, and a partial inhibited when DPI was applied in root (Figure 3A). Moreover, this inhibitor completely abolished the induction of SOD, GR, and CAT activities observed in leaves 30 min post inoculation (Figure 3B–D). Nevertheless, no significant differences were observed in the APX activity (Figure 3E).

**FIGURE 4:**
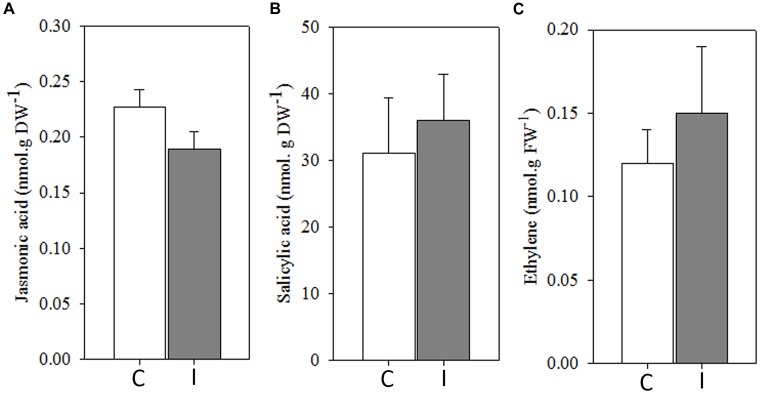
Systemic ROS generation precedes to systemic hormonal changes after root inoculation with *B. japonicum*. Jasmonic acid **(A)**, salicylic acid **(B)**, and ethylene **(C)** were quantified 30 min after root inoculation from leaves of non-inoculated (control, C) and inoculated (I) plants. Data are the means of four leaves ± SE for jasmonic acid and salicylic acid, and seven leaves for ethylene of three independent experiments. No significant differences were observed between control and inoculated plants (*p* < 0.05, Tukey test).

In addition, JA, SA, and ET levels remained apparently unaltered in leaves 30 min after root inoculation with *B. japonicum* (Figure 4).

### NFR5-Dependent Systemic ROS Induction After Root Inoculation

**FIGURE 5:**
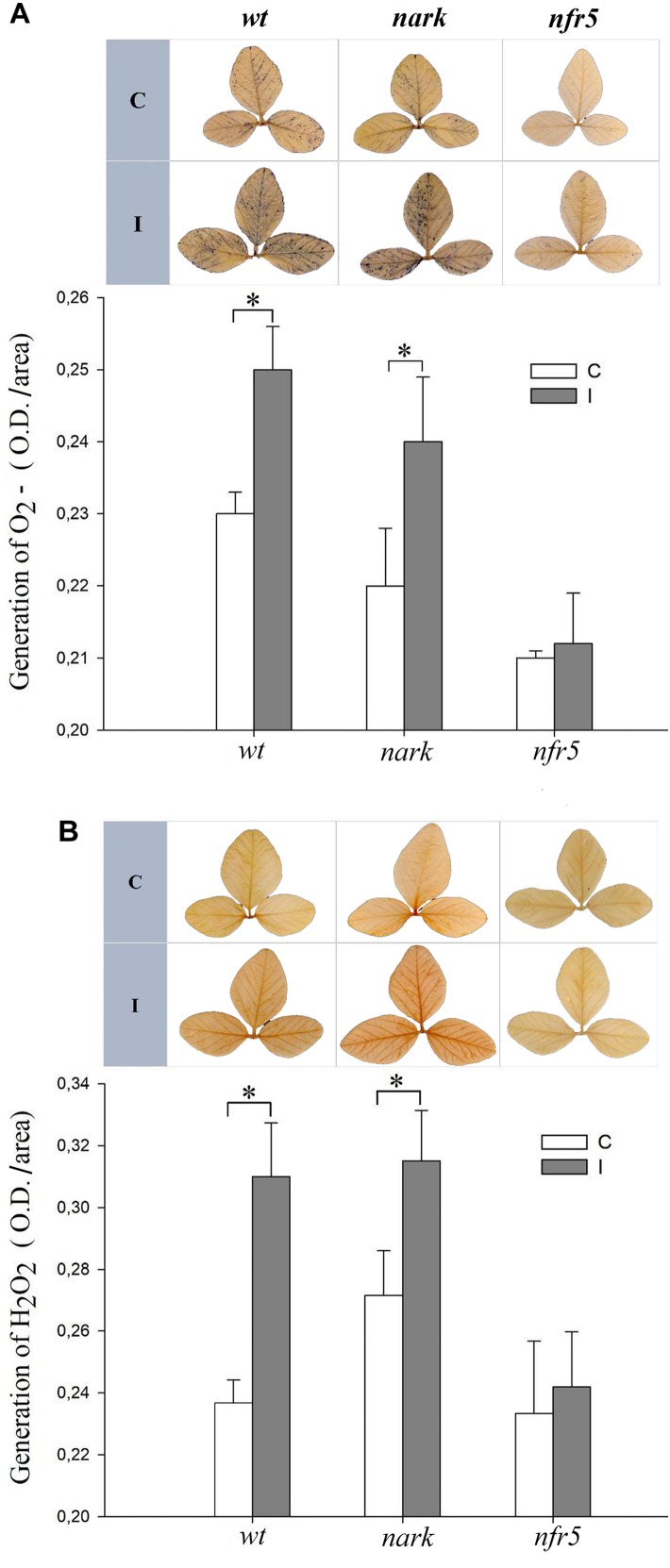
The systemic ROS induction after *B. japonicum* inoculation depends on NFR5 signaling pathways. Roots of 12-days-old *nfr5* and *nark*-soybean mutant plants or *wt* plants were inoculated with *B. japonicum* (I), and the first trifoliate leaf was sampled after 30 min and incubated with **(A)** nitroblue tetrazolium (NBT) or **(B)** 3,3′-diaminobenzidine (DAB) for O_2_^-^ and H_2_O_2_ staining, respectively. Non-inoculated plants were used as control (C). Upper panels, NBT and DAB stained leaves; lower panels, NBT and DAB quantification. The pictures of the stained leaves were transformed into 8-bit images and the gray value intensity (O.D.) was quantified with image processing software. The results are the means of three independent experiments (four leaves per treatment). Data are means ± SE. Asterisks indicate significant differences between control and inoculated plants (*p* < 0.05, Tukey test).

To further study the systemic redox changes induced during the soybean–*B. japonicum* interaction, we analyzed ROS generation in leaves of non-nodulating *nfr5-*mutant and hyper-nodulating *nark*-mutant soybean plants after 30 min of root inoculation (Figure 5). The leaves of *nark-*mutants showed a systemic ROS induction in response to *B. japonicum* inoculation similarly to that of the *wt* soybean plants (Figure 5). However, neither the systemic generation of H_2_O_2_ nor O_2_^-^ were induced in leaves of *nfr5-*mutant soybean plants 30 min post inoculation (Figure 5).

### From Root to Shoot: Root Inoculation Induced Systemic Tolerance to Photooxidative Stress

The legume–rhizobium symbiotic interaction can mediate an increased tolerance to abiotic stresses in host plants by an ISR modulated by JA and ET (Dimkpa et al., 2009; Pieterse et al., 2014). Furthermore, genetic and cell biological evidences revealed the role of ROS as a second messenger during the plant responses to the environment (Miller et al., 2007, 2008; Melchiorre et al., 2009). In this regard, the NADPH oxidase complex not only initiates ROS production but also amplifies them (Foreman et al., 2003; Torres and Dangl, 2005; Miller et al., 2009; Robert et al., 2009).

**FIGURE 6:**
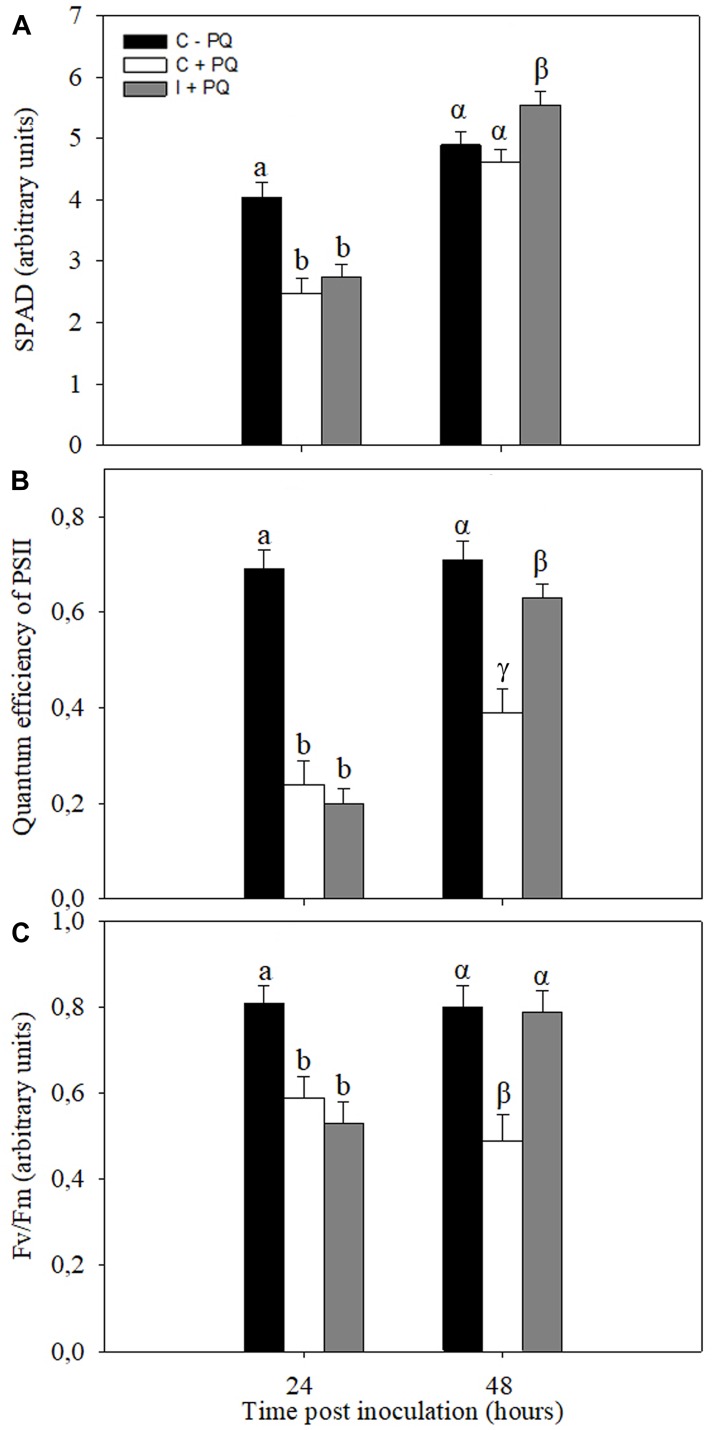
Root inoculation induced photooxidative stress tolerance in leaves. Paraquat (PQ; 20 μM) was sprayed in the first trifoliate leaf of inoculated plants after 24 and 48 h of root inoculation with *B. japonicum* (I +PQ). Non-inoculated plants were used as a control (C +PQ) and without PQ (C –PQ). The chlorophyll content (SPAD) **(A)**, quantum efficiently of the PSII (ΦPSII) **(B)**, and maximum quantum efficiency of Photosystem II (*F*_v_/*F*_m_) **(C)** were analyzed 24 h after PQ treatment. The results are the means of three independent experiments (nine leaves per treatment). Data are means ± SE. Different Latin and Greek letters indicate significant differences between treatments after 24 and 48 h post inoculation, respectively (*p* < 0.05, Tukey test).

Stress conditions, in general, are associated with increases in the ROS production, mainly in the chloroplasts, which ultimately leads to photooxidative stress. In order to evaluate the systemic beneficial effects of the symbiotic interaction, the tolerance of non-inoculated and inoculated plants to PQ treatments, an herbicide that increase the chloroplasts ROS production (Lascano et al., 2003, 2012; Melchiorre et al., 2009; Robert et al., 2009), were evaluated. The first trifoliate leaf of non-inoculated and inoculated plants was treated with PQ at 24 and 48 h post inoculation. The selected physiological parameters related to photosynthesis to be assessed after 24 h of PQ application were SPAD, PSII, and *F*_v_/*F*_m_ (Supplementary Figure S2). Under control conditions (without PQ), no significant differences were observed between non-inoculated and inoculated plants (Supplementary Figure S3). Likewise, no significant differences were observed between non-inoculated and inoculated plants treated with PQ at 24 h after root inoculation (Figure 6). However, the leaves of inoculated plants were more tolerant than leaves of non-inoculated plants when PQ was applied after 48 h of root inoculation (Figure 6).

### ISR/PGPR-Like Response: Participation of Nod-Factors and AON-Signaling Pathways?

**FIGURE 7:**
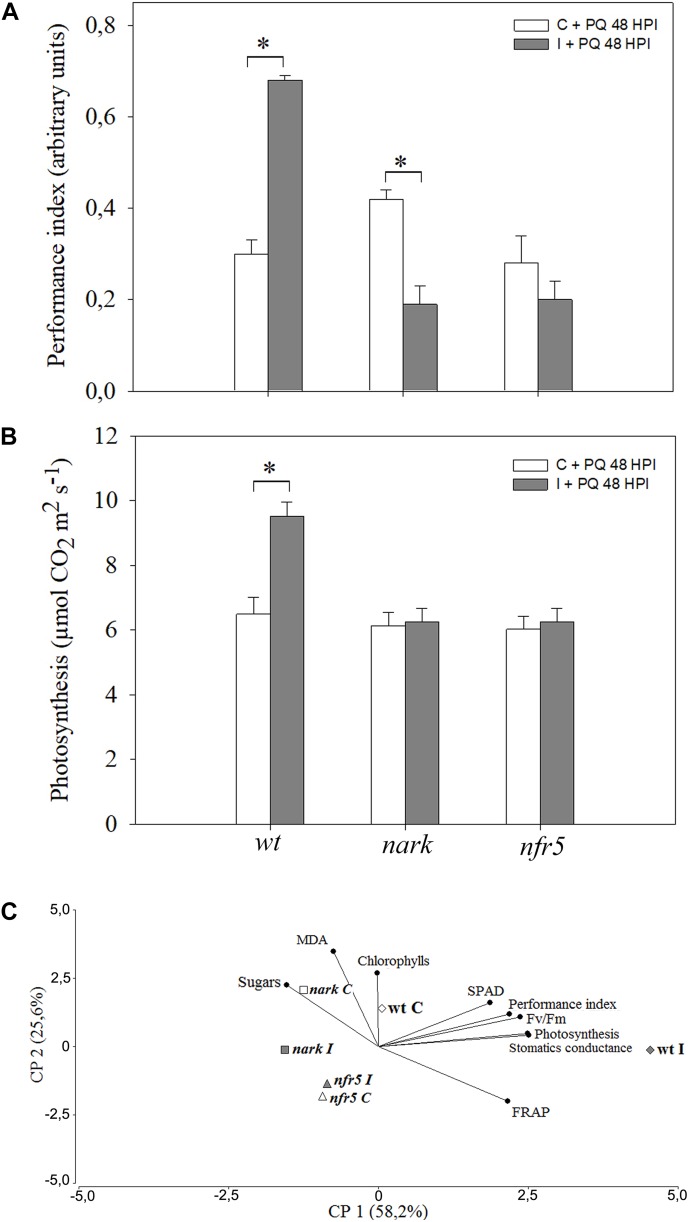
The induced-photooxidative stress tolerance in leaves after *B. japonicum* inoculation depends on Nod factors- and AON-signaling pathways. The first trifoliate leaf of 12-days-old *nfr5*- and *nark*-mutant soybean plants and *wild type* (*wt*) plants were sprayed with 20 μM paraquat (PQ) after 48 h of root inoculation with *B. japonicum* (I). Non-inoculated plants were used as a control (C). The chlorophyll content, SPAD, maximum quantum efficiency of Photosystem II (*F*_v_/*F*_m_), sugars and MDA content, photosynthesis, stomatic conductance, performance index and the ferric reducing ability of plasma (FRAP) were analyzed 24 h after PQ treatment. **(A)** Performance index (PI) was set as an indicator of the overall fitness of the photosynthetic apparatus. **(B)** Net photosynthesis. Data are means ± SE of nine leaves of three independent experiments. Asterisks indicate significant differences between control and inoculated plants (*p* < 0.05, Tukey test). **(C)** Biplot from the first and second principal components (PC1 and PC2) of principal components analysis (PCA) showing relationships between inoculated *wt* plants and SPAD; Performance Index; *F*_v_/*F*_m_; Photosynthesis; Stomatic conductance and FRAP.

To evaluate the involvement of Nod-factors and AON signaling in an ISR/PGPR-like response induced during soybean–*B. japonicum* interaction, *nrf5*- and *nark*-mutant soybean plants were used. Leaves of mutant and *wt* plants were treated with PQ 48 h after root inoculation, and then diverse physiological–biochemical parameters were assessed (Figure 7). The performance index is an integrative and sensitive parameter that gives quantitative information on the plant performance reflecting the functionality of both photosystems I and II (Strasser et al., 2000). After PQ treatments, leaves of *wt* inoculated plants showed an increase in the performance index in comparison to leaves of non-inoculated *wt* plants (Figure 7A). However, the leaves of inoculated and non-inoculated *nfr5-*mutant plants showed no significant differences after this treatment (Figure 7A). Curiously, the *nark-*mutant plants showed a lower tolerance upon inoculation (Figure 7A). Furthermore, the photosynthesis rates were significantly lower in *wt* non-inoculated with respect to *wt* inoculated plants, whereas no effect of inoculation was observed in leaves of *nfr5-* and *nark-*soybean mutants (Figure 7B). In a PCA performed to identify associations between physiological–biochemical parameters and the different plant genotypes under photooxidative stress treatments, the first two principal components (PC 1 and PC 2) of the analysis explained 85.1% of total variability in the data (Figure 7C). In a PCA, the cosine of the angle between two parameter vectors approximates the association among the parameters, with acute and obtuse angles indicating positive and negative correlations, respectively, and right angles denoting no correlation between parameters. The PC1 of the biplot indicated that SPAD, performance index, *F*_v_/*F*_m_, photosynthesis, stomatic conductance and FRAP were positively associated and these vectors were oriented toward *wt*-inoculated plants (Figure 7C). Interestingly, control and inoculated *nfr5*- and *nark*-mutant genotypes were located closed to non-inoculated *wt* plants (Figure 7C).

**FIGURE 8:**
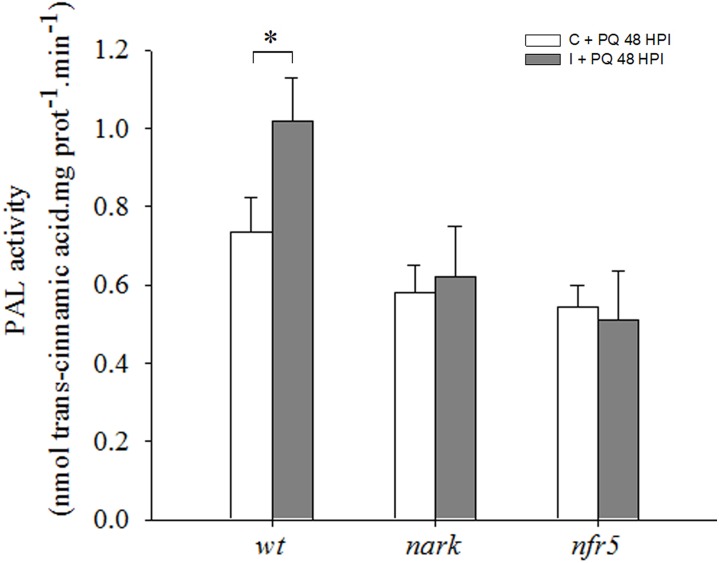
Phenylalanine ammonia-lyase (PAL) is induced under photooxidative stress conditions in *wild type* (*wt*) inoculated plants. The first trifoliate leaf of 12-days-old *nfr5*- and *nark*-mutant soybean plants and *wt* plants was sprayed with 20 μM paraquat (PQ) after 48 h of root inoculation with *B. japonicum* (I), non-inoculated plants were used as a control (C). PAL activity was measured in the first trifoliate leaves after 24 h of PQ treatment. Data are means ± SE of three leaves of three independent experiments. Asterisks indicate significant differences between comparisons (*p* < 0.05, Tukey test).

Phenylalanine ammonia lyase activity, a key enzyme of the phenylpropanoid pathway and another ISR marker, was evaluated. The analysis of PAL activity revealed that this activity was induced in leaves of *wt* inoculated plants as compared to *wt* non-inoculated, while no significant differences were observed between non-inoculated and inoculated *nfr5-* and *nark-*mutant plants (Figure 8).

Likewise, to analyze the participation of hormones related to ISR or AON, we assessed the levels of JA and SA in leaves after 72 h of root inoculation (Supplementary Figure S4). The content of these hormones was similar among treatments and genotypes (Supplementary Figure S4).

## Discussion

The relationships between plants and microorganisms are active, co-evolutionary processes that can result in incompatible or compatible interactions. The great majority of microorganisms are not able to invade and establish compatible interaction with plants, whereas a few species have developed strategies to establish a compatible interaction ranging from mutualistic (symbiotic) to pathogenic interactions. Plants recognize different signal molecules from microorganisms, like MAMPs and other pathogenic and symbiotic signals that modulate the immune system of plants (Limpens et al., 2015; Couto and Zipfel, 2016). The ligand molecules, receptors and the downstream signaling pathways involved in plant–microorganism interaction have been deeply characterized in the last 10 years (Zipfel and Oldroyd, 2017). In this sense, the species-specific legume rhizobium symbiotic interactions and race-specific plant pathogen interactions share many similarities (Cao et al., 2017). MAMPs and symbiotic signals are recognized by cell-surface receptor kinases, which are composed by an extracellular domain involved in ligand perception, a transmembrane domain, and an intracellular kinase domain (Gourion et al., 2015; Miwa and Okazaki, 2017; Masson-Boivin and Sachs, 2018).

Legume rhizobium symbiotic interactions are orchestrated by a fine-tuning molecular dialog. Initially, the recognition of both Nod factors and MAMPs involves different cellular events, including increases of ROS production and cytosolic calcium concentration. Previously, we characterized the local redox changes and calcium involvement in root hairs during soybean–*B. japonicum* symbiotic interaction under control, abiotic and biotic stress conditions. A specific symbiotic calcium-dependent ROS signature occurred during the early events of soybean–*B. japonicum* symbiosis, which was completely different to ROS signature induced by pathogen elicitors or by abiotic stressful condition during the symbiotic interaction (Muñoz et al., 2012). Under moderate short-term salt stress (50 mM NaCl) treatment, *B. japonicum* could be sensed as a pathogen and its sensing may induce root hair cell death by a hypersensitive-like response and therefore may inhibit the nodulation process (Muñoz et al., 2012; Robert et al., 2014, 2018). Interestingly, the addition of calcium partially rescued the root hair death and inhibition of nodulation (Muñoz et al., 2014a). These results are in line with recent study that showed close similarities between symbiotic an immune pathway (Zipfel and Oldroyd, 2017).

In addition to generating local responses in roots, plant microorganism interactions also induce systemic responses that prepare the whole plant to upcoming challenges. SAR and ISR are the most studied systemic responses triggered by pathogen or beneficial microorganism, respectively. These systemic responses are mainly modulated by SA, for SAR, and JA and ET, for ISR. On the other hand, SAA is a systemic response triggered by abiotic stress conditions that provoke excess excitation energy, where chloroplastic and apoplastic ROS production play a key role.

Even when some evidence demonstrates the role of ROS as systemic signals (Miller et al., 2009; Gilroy et al., 2014), this aspect has not been investigated in legume–rhizobium interaction. The initial aim of the present work was to characterize the systemic redox changes induced during the soybean–*B. japonicum* symbiotic interaction and to assess their relationship with ISR/PGPR-like response. Our results showed that soybean–*B. japonicum* interaction induces a rapid redox systemic changes given by a transient peak of ROS at 30 min post inoculation (Figure 1). Furthermore, this interaction produces changes in the total content of ascorbate, glutathione redox state as well as higher MDA content (Figure 2), and higher antioxidant enzymes activity (Figure 3). Ascorbate-glutathione-NADPH and the interconnected antioxidant enzymes of the Asada–Halliwell cycle are key components of the redox hub that controls the cellular redox state. Perturbations of this redox buffering mechanism trigger redox signaling pathways, where even the oxidative damage could be also part of the oxidative or redox signaling, explaining the dual role of ROS as toxic and signal molecules. Ascorbate and glutathione are the major soluble antioxidants. The redox potential among NADPH, glutathione and ascorbate, where glutathione has middle values between NADPH and ascorbate could explain why we found changes in the redox state only in glutathione pool. Moreover, many evidences indicate that conditions with enhanced ROS production has less impact on the ascorbate redox state pool than on glutathione pool, generating altered cellular redox state characterized by a highly reduced state of the ascorbate pool and partially oxidized state of the glutathione pool. Changes in the glutathione redox state have been previously characterized in oxidative signaling processes (Kranner et al., 2006; Foyer and Noctor, 2011). The cellular redox state, mainly determined by the ratio between generation and scavenging capacity of ROS in different subcellular compartments, is an integral source of information for the plant cell that modulates growth, development and different responses to environmental conditions (Foyer and Noctor, 2011). Recently, systemically auto propagating waves of ROS, calcium and electric signals has been integrated in a model for rapid systemic cell-to-cell communication in plants that is involved in the acclimation to abiotic stress condition (Choi et al., 2017). In this regard, the NADPH oxidase complex (RBOH respiratory burst homolog protein) plays a key role within this model. The RBOH proteins are localized on the plasma membrane and generate superoxide radical in the apoplast, which quickly dismutates to hydrogen peroxide spontaneously or by SOD activity.

Our results also showed that the inhibitor of NADPH oxidase complex, DPI, abolished the systemic redox changes induced during the soybean–*B. japonicum* symbiotic interaction, indicating the involvement of this complex in the systemic redox signaling (Figure 3).

**FIGURE 9:**
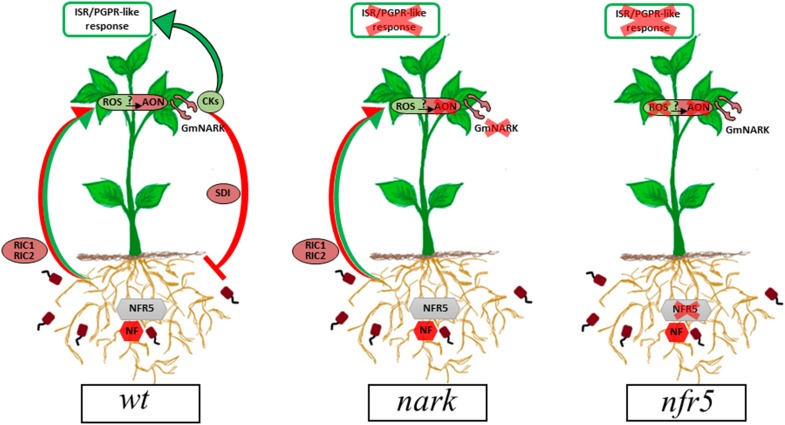
Proposed model for the ISR/PGPR-like response induced during *B. japonicum*–soybean interaction. Red arrows and circles indicate the already described signaling pathway involved in AON. Green arrows and circles indicate the proposed signaling pathways arising from this research. In *wt* plants, rhizobia-induced CLE peptides (RIC1 and RIC2) are produced in root after NFR5-dependent symbiont perception. Then, CLE peptides are transported via xylem to shoot, where they bind to GmNARK triggering the production of the SDI, which moves back to the roots and inhibits nodule formation (red arrows and circles). A potential role for the systemic ROS production in the AON mechanism as well as in the ISR/PGPR-like response is proposed. In this sense, cytokinins play a dual role, participating in the mechanism involved in AON and in the ISR/PGPR-like response (green arrows and circles). AON, autoregulation of nodulation; CKs, cytokinins; GmNARK, *Glycine max* nodulation autoregulation receptor kinase; ISR, induced systemic resistance; NF, Nod factors; NFR5, Nod factor receptor 5; PGPR, plant growth-promoting rhizobacteria; ROS, reactive oxygen species; RIC, rhizobia-induced CLE peptides; SDI, shoot-derived inhibitor.

As it was mentioned above, ROS and redox changes could act as priming molecules and thus improve the responses to different stress conditions. In line with this, inoculated soybean plants were more tolerant to a photooxidative stress condition induced by PQ treatments. This finding indicates that *B. japonicum* inoculation could prime soybean plants by inducing ISR/PGPR-like response through redox changes (Figure 5–7, 9).

To further investigate the specificity of the systemic response and its role in the ISR/PGPR-like response, we performed treatments with purified Nod-factors from *B. japonicum* as well as using Nod factor receptor plant mutants (Figure 5, 7 and Supplementary Figure S1). Similarly to *B. japonicum* inoculation, Nod factors treatment induced transient peak of ROS. Moreover, the systemic response was completely different when plant roots were treated with two derivatives of chitin oligosaccharides, Nod factors or chitosan, a potent pathogen elicitor (Supplementary Figure S1).

The Nod factor receptors have been characterized in different legumes. They are composed by pairs of LysM-receptor kinases and therefore share many similarities to chitin receptors. Soybean contains two genes GmNFR5α and GmNFR5β among others within its genome but only GmNFR5α is functional in *Glycine max*. Chemically induced non-nodulating nod139 mutant (Carroll et al., 1986) present a single nucleotide substitutions (non-sense) within the coding region of the GmNFR5α and this results in the elimination of most of the kinase domain (Indrasumunar et al., 2010). The *B. japonicum* inoculation of *nfr5-*mutant (nod139) lacked the systemic ROS changes and was non-tolerant to photooxidative stress conditions through ISR/PGPR-like response (Figure 5, 7, 9).

In this study, we also analyzed the participation of AON in the systemic redox changes and ISR/PGPR-like response induced by *B. japonicum* inoculation. For this purpose, we used mutant plants that lost the GmNARK function (nts1007 with a mutation of V370D) and that exhibit supernodulation phenotypes (Searle et al., 2003; Lin et al., 2010). GmNARK is an LRR receptor kinase homolog to *Arabidopsis thaliana* CLAVATA1, but without conserved function of inflorescence meristem regulation. GmNARK is expressed in the root and shoot and its main function is in local and systemic regulation of nodulation. Locally, root GmNARK plays a key role in the nitrate inhibition of nodulation mediated by NIC (Lim et al., 2011; Reid et al., 2011a, 2013; Mirzaei et al., 2017; Ferguson et al., 2018). Systemically, leaf GmNARK participates in the AON by perceiving CLE peptides from rhizobium inoculated roots (RIC1 and RIC2) and inducing the synthesis of SDI, which is transported by phloem to the root where it provokes the inhibition of nodulation (Searle et al., 2003; Lim et al., 2011; Reid et al., 2011b; Ferguson et al., 2018). The *nark*-mutant showed the same systemic redox changes than the *wt* plants (Figure 5); however, this mutant failed to induce the ISR/PGPR-like response with tolerance to photooxidative stress condition (Figure 7, 9). Likewise, the phenylalanine ammonia-lyase (PAL) activity, which catalyzes the conversion of L-phenylalanine to *trans*-cinnamic acid, showed a significant increase only in the inoculated *wt* plants, whereas this activity remained unaltered in both mutants upon the inoculation (Figure 8). These results indicate that both Nod factors perception and AON signaling are necessary for the activation of PAL, the key enzyme in the phenylpropanoid pathway and a marker of ISR and primed plants (Conrath, 2011).

Transcriptomic analysis using this mutants showed that GmNARK regulates the expression of JA pathway genes and participates in plant defense responses (Kinkema and Gresshoff, 2008). The promoter region of GmNARK gene contains multiple *cis*-elements involved in plant responses to hormones and biotic stresses. Likewise, GmNARK expression is induced by ABA and NaCl and its overexpression increases the sensitivity to salt and ABA treatment, thus indicating the involvement of this receptor also in the responses to abiotic stresses (Cheng et al., 2018). Pieterse et al. (2014) have well established the importance of SA, JA/ET as key signals in the regulation of the plant systemic immune responses SAR and ISR. In the present work, these hormones neither showed any difference in *wt* soybean at 30 min post inoculation (Figure 4), where the ROS peak was detected, nor at 72 h post inoculation in *wt, nark* and *nfr5* (Supplementary Figure S4), where the ISR/PGPR-like response is established.

The molecular identity of SDI is still unknown. In *Lotus japonicum* the CLE-RS1/2-HAR1 interaction triggered the production of CKs in shoot, which have been postulated as SDI. CKs production in the shoot of *Lotus* is mediated by the activation of isopentenyl transferase 3 (LjIPT3) in a HAR1 dependent pathway (Sasaki et al., 2014). Likewise, CKs have been largely characterized as positive regulator of nodulation at root level (Murray et al., 2007). This dual role could depend on the site of synthesis, the concentration and the type of CKs as well as the receptor involved in the process. Moreover, the CKs that promote nodulation are independent of AON signaling. However, the anti-senescence and stress tolerance functions of CKs induced during legume–rhizobium symbiotic interaction have not been studied yet. In this context, we propose that ISR/PGPR-like response induced during legume–rhizobium symbiotic interaction may be related to the AON-related CKs (Figure 9).

After root inoculation, and in opposition to what was observed in *Lotus*, recent data showed an GmNARK-independent induction of GmIPT5, an ortholog of LjIPT3 in soybean shoots (Mens et al., 2018). This finding suggests differences between soybean and *Lotus* pathway or an uncertainty of the IPT-AON association.

Altogether, our results showed that the NADPH-dependent transient systemic redox changes induced after root inoculation with *B. japonicum* in soybean rely upon Nod factor signaling and that these changes are involved in the priming and induction of the ISR/PGPR-like response (Figure 9). In this sense, CKs play a dual role, participating in the mechanism involved in AON and ISR/PGPR-like response (Figure 9). Finally, the involvement of the AON mechanisms in the ISR/PGPR-like response also suggests that systemic redox changes induced during soybean–*B. japonicum* interaction are early components of the AON pathway. Nevertheless, future studies should be addressed to confirm this hypothesis.

## Author Contributions

RL, SA, and NM designed the experiments. TF-G and RD conducted the experiments. TF-G analyzed the data. TF-G, GR, and RL co-wrote the manuscript.

## Conflict of Interest Statement

The authors declare that the research was conducted in the absence of any commercial or financial relationships that could be construed as a potential conflict of interest.

## Supplementary Material

FIGURE S1 Systemic H_2_O_2_ generation in leaves after root treatments with *B. japonicum*, purified Nod factors and chitosan. H_2_O_2_ was quantified by spectrofluorometer. The results are the means of three independent experiments (six leaves per treatment). Data are means ± SE. Asterisks indicate significant difference between non-inoculated and inoculated plants (*p* < 0.05, Tukey test).

FIGURE S2 Diagram of the experimental system for paraquat (PQ) treatment. 12-days-old soybean plants were inoculated with *B. japonicum*, and PQ treatments were performed in leaves at 24 and 48 h post inoculation (hpi). The physiological parameters were measured 24 h after PQ treatment.

FIGURE S3 Relatives values (inoculated/control) of SPAD, PSII and *F*_v_/*F*_m_ in plants at 24 and 48 h without paraquat (PQ) treatments; the parameters were measured at 48 and 72 h post inoculation, respectively. The results are the means of three independent experiments (nine leaves per treatment). Data are means ± SE. No significant differences were observed between non-inoculated and inoculated plants (*p* < 0.05, Tukey test).

FIGURE S4 Jasmonic acid **(A)** and salicylic acid **(B)** contents in leaves of non-inoculated and inoculated plants at 72 h post inoculation (hpi). The results are the means of four replicates ± SE (*p* < 0.05, Tukey test). No significant differences were observed between non-inoculated and inoculated plants.

## Abbreviations

ABA: abscisic acid
AON: autoregulation of nodulation
APX: ascorbate peroxidase
CAT: catalase
CKs: cytokinins
DPI: diphenyleneiodonium
ET: ethylene
GmNARK: *Glycine max* nodulation autoregulation receptor kinase
GR: glutathione reductase
hpi: hours post inoculation
HAR1: hypernodulation aberrant root formation 1
IPT: isopentenyl transferase
ISR: induced systemic resistance
JA: jasmonic acid
LCO: lipo-chitooligosaccharide
LRR: leucine-rich repeat
MAMPs: microorganism associated molecular patterns
MDA: malondialdehyde
NF: Nod factors
NFR5: Nod factor receptor 5
NIC: nitrate-induced CLE peptides
PAL: phenylalanine ammonia-lyase
PGPR: plant growth-promoting rhizobacteria
PQ: paraquat
RBOH: respiratory burst oxidase homolog
RIC: rhizobia-induced CLE peptides
ROS: reactive oxygen species
SA: salicylic acid
SAA: systemic acquired acclimation
SAR: systemic acquired resistance
SDI: shoot-derived inhibitor
SOD: superoxide dismutase
