# Supplementary figures and images for: Redox Systemic Signaling and Induced Tolerance Responses During Soybean–Bradyrhizobium japonicum Interaction: Involvement of Nod Factor Receptor and Autoregulation of Nodulation
Source: Front Plant Sci. 2019 Feb 15;10:141. doi: 10.3389/fpls.2019.00141 (PMC6384266; doi:10.3389/fpls.2019.00141)

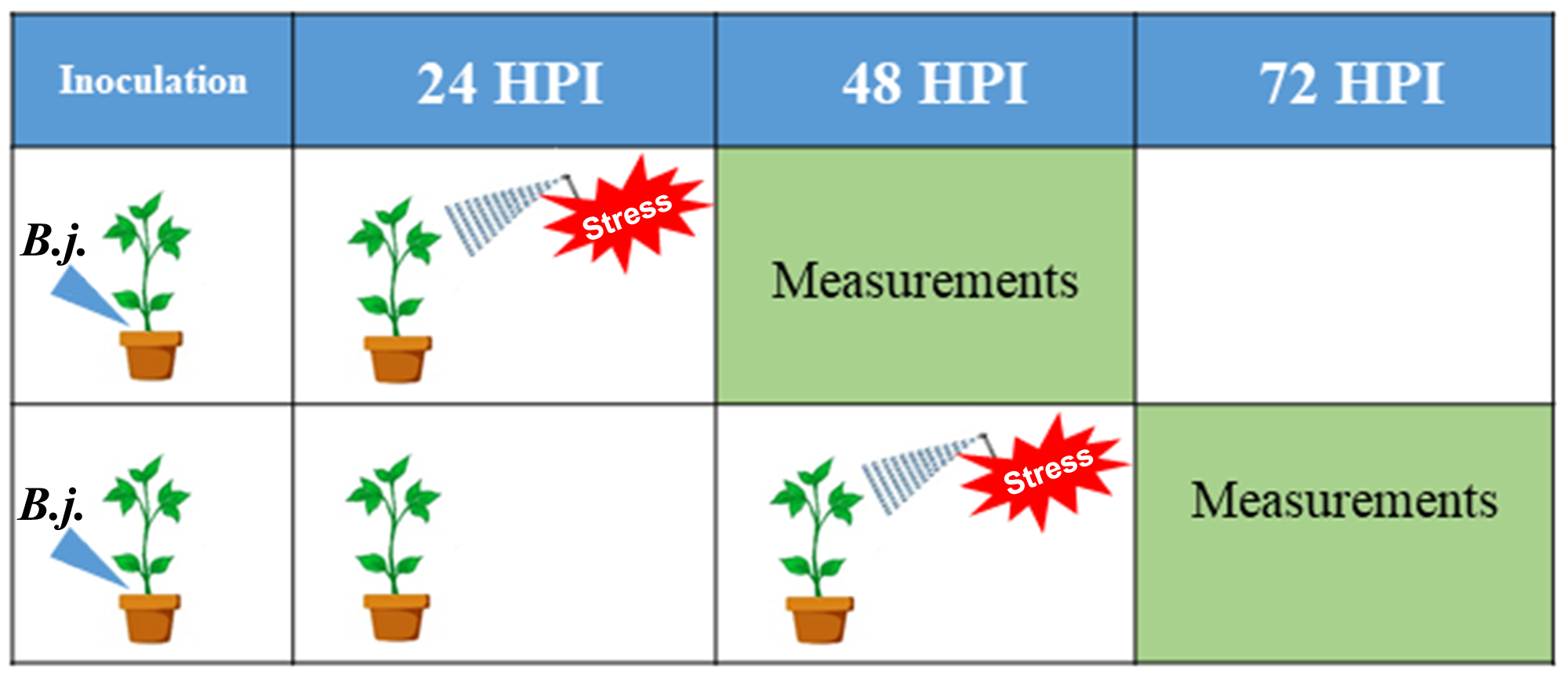

Supplement: FIGURE S2 — Diagram of the experimental system for paraquat (PQ) treatment. 12-days-old soybean plants were inoculated with B. japonicum, and PQ treatments were performed in leaves at 24 and 48 h post inoculation (hpi). The physiological parameters were measured 24 h after PQ treatment. [file Image_2.tif]

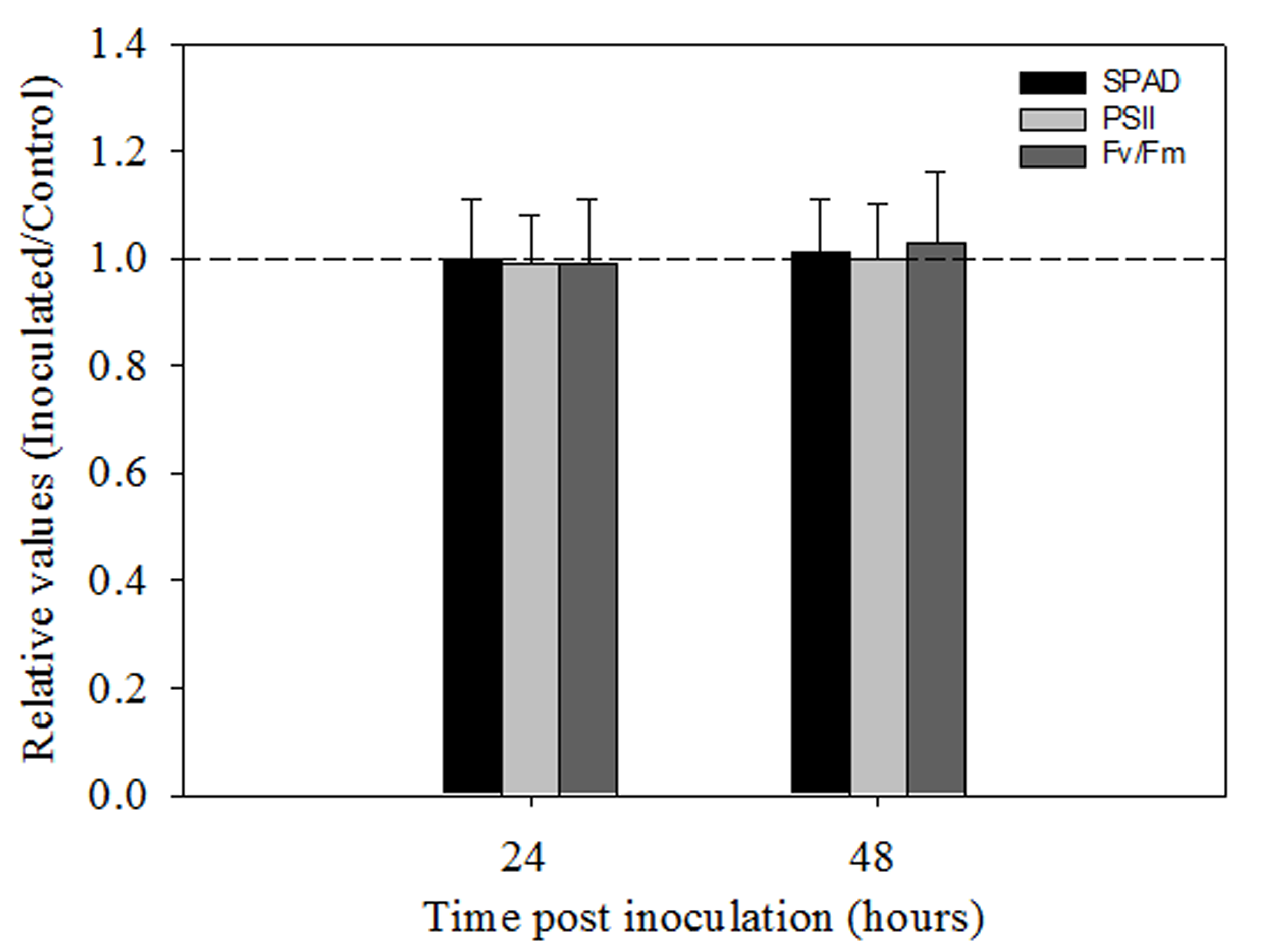

Supplement: FIGURE S3 — Relatives values (inoculated/control) of SPAD, PSII and Fv/Fm in plants at 24 and 48 h without paraquat (PQ) treatments; the parameters were measured at 48 and 72 h post inoculation, respectively. The results are the means of three independent experiments (nine leaves per treatment). Data are means ± SE. No significant differences were observed between non-inoculated and inoculated plants (p < 0.05, Tukey test). [file Image_3.TIF]

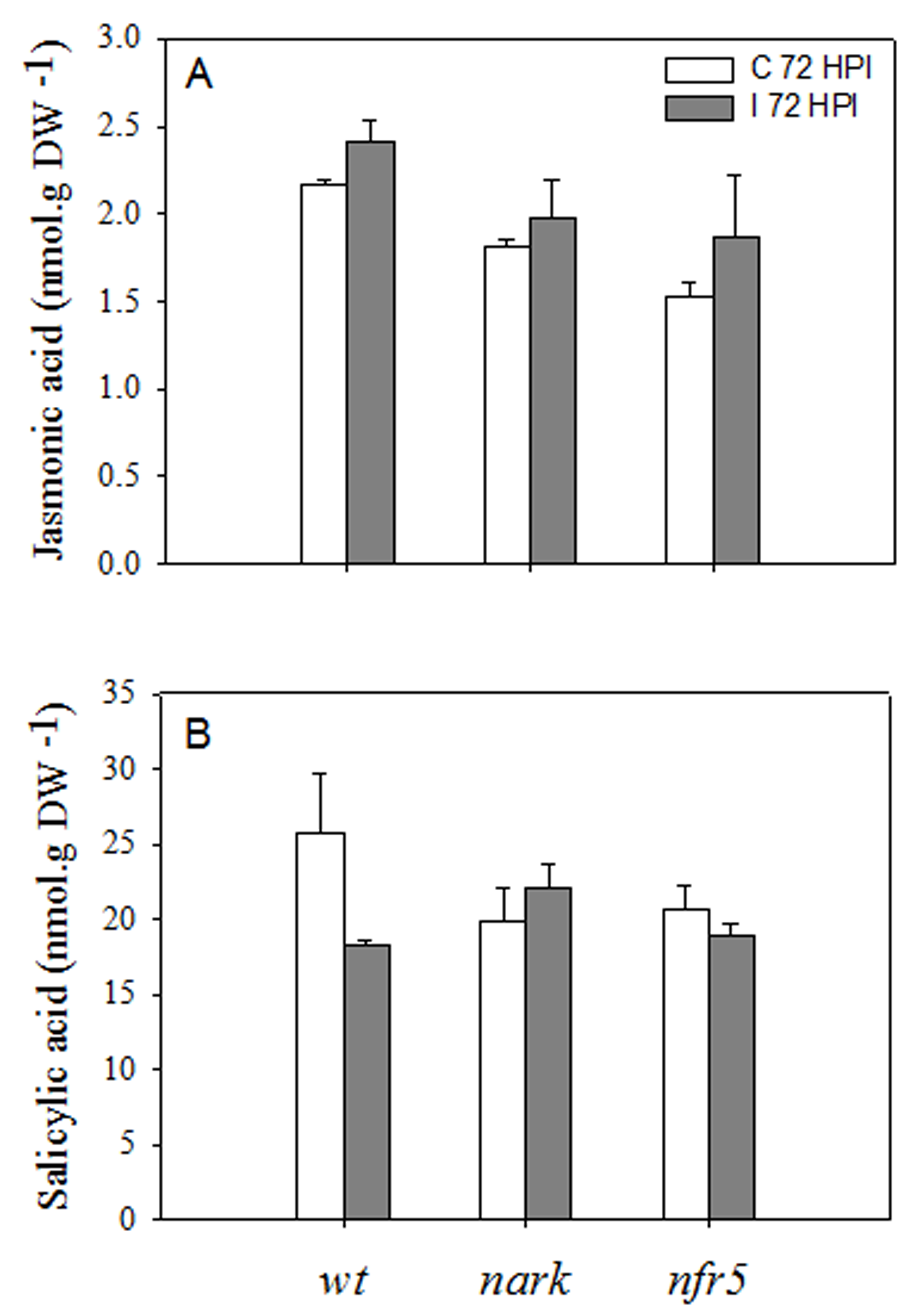

Supplement: FIGURE S4 — Jasmonic acid (A) and salicylic acid (B) contents in leaves of non-inoculated and inoculated plants at 72 h post inoculation (hpi). The results are the means of four replicates ± SE (p < 0.05, Tukey test). No significant differences were observed between non-inoculated and inoculated plants. [file Image_4.TIF]
